# Contributions to Surgery

**Published:** 1842-03-12

**Authors:** Reynell Coates


### MEDICAL EXAMINER.

NEW SERIES.

No. 11.] PHILADELPHIA, MARCH 12, 1842. [Vol. I.

### *Contributions to Surgery.*

BY REYNELL COATES, M. D.

*Additional note on the applications of the bran dressing.*—In a recent conversation with Dr. John Rhea Barton on this subject, consequent upon the remarks published in the ninth number of this Journal, that gentleman reminded us of some facts of importance which cannot be uninteresting to our readers. He was originally led to the employment of the bran in cases of compound fracture accompanied with extensive laceration, where an equable support without the uncertain pressure of bandages became a most important object. It is well known that such cases are peculiarly prone to produce mortification, and that any dressing offering the least restraint to the local circulation greatly enhances the danger. Rest—absolute rest—and not pressure, is the chief indication. But the swelling attendant on the subsequent inflammation inevitably occasions swelling; and the most cautiously applied bandage, however slight may be the restraint which it produces in the first instance, speedily becomes tightened by the increasing bulk of the parts, and undoubtedly promotes the occurrence of gangrene in many instances. It is chiefly for the purpose of readily adapting the dressing to this change in dimensions, and to accomplish this with the least possible agitation of the limb, that the bandage of Scultetus, or the less perfect ordinary bandage of strips is now almost universally preferred to the common roller in the treatment of compound fractures. But even the bandage of Scultetus can not be applied without exerting considerable pressure, and thus enhancing, to a certain extent, the danger of mortification; while the gradual or more rapid exudation of blood from vessels too small or too deeply seated to be properly secured by ligature, moistens the strips, increasing the rigidity of the dressings, and frequently rendering it impossible to modify their action without agitating the limb in a painful and, it may be, dangerous manner. Again: When the laceration is extensive, this very exudation of blood from broad surfaces and minute vessels often endangers the life of a patient already almost in a state of collapse from the shock of the accident. It must be arrested—and this can only be accomplished by *pressure.* It is, therefore, of the utmost importance that we should so regulate this dangerous resource as to render its action as mild and as purely local as possible.

The first case in which Dr. Barton employed the bran dressing, was of the character just described, and he observed that the blood escaping from the wounds gradually amalgamated the bran in the immediate neighbourhood of the injury into a soft, but solid mass, which, by its increasing extent, and its enlargement by the absorption of fluid, continually meets with more and more resistance from the dry bran which surrounds it, and consequently reacts directly on the wounded surfaces with perpetually accumulating force, until it arrests the hemorrhage; when the process instantly ceases. Thus we have an exceedingly bland application—a mixture of bran and coagulated blood—acting in a manner purely local, without the slightest embarrassment to the general circulation of the injured limb, and one which cannot exert any degree of pressure beyond the precise necessities of the case.

It frequently happens, in the progress of reaction, that a hemorrhage of this character is renewed by the returning vigour of the circulation. In this case, the compress, if we may so style it, again becomes gradually enlarged, until the pressure is sufficient once more to check the flow.

This beautiful process has been very frequently employed by the able and ingenious surgeon with whom it originated, and always with the happiest effects. It escaped our recollection when penning the former note, but the subject deserves another remark.

We have spoken of the facility with which the bran dressing may be shifted to permit an examination of the injured parts; but, in extensive lacerations, such examinations should never be lightly made at a moment too early after the accident.

The limb being placed in a suitable fracture box, the broken bones adjusted, and an easy attitude secured, it should be covered completely, and to a considerable depth. It would then be folly to disturb the bran for several days, in consequence of any slight change in the position of the fragments;—(and a great one can never occur in a well regulated apparatus,)—for, such changes are of very little importance during the first days of a severe compound fracture, and our interference would endanger the recurrence of serious hemorrhage. When suppuration is fully established, such an accident, though not absolutely impossible, is extremely rare, because the wound is then completely covered with a new secreting membrane foreclosing the torn capillaries. Scarcely ever will occasion warrant an examination in less than four or six days, except when the occurrence of extensive gangrene is plainly indicated by the condition of the patient and the appearance of the limb at a distance from the seat of injury. Any accidental malposition of the fragments which can occur under skilful treatment, may be corrected after such an interval as readily as at an earlier moment.

*Note on the treatment of muscular spasm as an opponent to immediate reduction in fractures of the inferior extremities.*—Fortunately, as we think, for the interests of the sufferers, the extended position is now almost universally preferred in the treatment of fractures of the lower extremities on this side of the Atlantic. The plan of laying the limb upon a pillow, while the patient reclines on his side, after the manner of Pott, has been long discarded. The double inclined plane of White, with its hundred modifications, is very seldom heard of, except where it is employed in fractures of the leg; the lower portion of the plane being then supported in a horizontal or slightly more elevated attitude, for the simple purpose of favouring the return of blood, in those cases in which the obliquity of the fracture does not render necessary the exercise of permanent extension. The simplest mode of constructing this apparatus is of course the best, and, in most cases of fracture of the leg, unattended with shortening of the limb, the proper elevation of the ordinary fracture box, upon a frame or other support placed on the bed, secures all the advantages of the multiform contrivances constructed upon the plan of White, without their disadvantages; for, there is no necessity for giving support to the thigh unless the fracture be seated very near the knee, or the contusions or wounds complicating the case extend to the neighbourhood of the ham. Resort was had to something like this method in the case reported by Dr. C. Hartshorne, page 134.

In fractures of the thigh, three forms of apparatus are now almost exclusively employed among surgeons of the school of Philadelphia, namely, "The modification of Dessault," by Dr. Physick; "The modification of Boyer," by Dr. Hartshorne; "The modification of Hagedorn," by Dr. Gibson. In addition to these, we occasionally, but rarely, meet with the apparatus of Amesbury, and very recently, the modification of the old Arabian method of "the immovable apparatus" threatens to become, unfortunately, fashionable with some in the treatment of all fractures. To each of these we propose to devote a note in future numbers, perhaps extending our comments over a wider field. At present, and as a preliminary matter it is proper to comment upon the causes of muscular spasm in fractures, and the mode of avoiding or alleviating it.

Local cramps,—by which is meant such as are not symptomatic of irritation of the small intestines, or of nervous affections seated at a distance—may be occasioned either by a partial or undue contraction of a portion of a muscle not extended to the whole organ, or by direct irritation of a muscle or its motor nerve. Contusions very rarely produce cramps, and lacerations—if not extensive—quite as rarely, unless tetanic spasms be classed under the same head. Amputations, on the contrary, are generally followed by such symptoms in greater or less severity;—a consequence obviously the result, in most instances, of the distruction of muscular attachments, permitting the fibres to undergo irregular and partial contractions.

Great distress is often experienced, from cramps, in fractures accompanied by shortening of the injured limbs, and many cautions have been given with regard to the gradual manner in which extension should be effected in order to avoid the production of the evil. But, let us inquire what are the conditions favourable to cramp in such cases.

The shortening of the distance between the origin and insertion of the muscles necessarily permits the irregular action of their fibres; and hence, the muscular trembling so often noticed in the thigh before complete reduction—a trembling precisely similar to that observed in the stump after amputation. This appears to us the chief cause of the spasms. The next cause—scarcely less important—is the direct irritation of the jagged or sharp extremities of the fragments pressing upon the neighbouring muscles or nerves which may or may not be lacerated. The too exclusive consideration of the latter cause is the probable reason why the prudent direction of Boyer, to bring the limb to the full length *gradually*, has led so many surgeons to proceed in an exceedingly dilatory manner in effecting extension or counter-extension, lest the force employed should provoke spasms of the muscles. The same danger has been urged by the disciples of Pott, in favour of his favourite doctrine of the semi-flexed position, now, fortunately, becoming less numerous even in England.

But, who ever heard of treating an ordinary cramp, in an uninjured patient, by relaxing the affected muscle? Is not the organ almost instinctively extended and pressed upon firmly at the moment, and is not this proceeding generally productive of immediate relief? Certainly, placing the limb in a flexed position must necessarily increase the danger of spasm in a fractured thigh; for it is in the flexors of the leg, and the psoas and illiacus muscles that cramp usually occurs; and, in all these the flexed position lessens the distance between the attachments and facilitates irregular action.

But, with regard to spasm occasioned by the direct irritation of the fragments pressing upon the injured nerves or muscles: Is not that a most commendable philosophy which teaches us to leave those fragments out of place for a long time, in order to remove a difficulty caused solely by their displacement? Some surgeons allow three days for the *gradual* reduction of a fractured thigh with shortening; some prefer a week, and one justly distinguished practitioner formerly recommended two weeks as a suitable time. Now, in addition to the fact that, during this delay, the muscles are gradually but steadily accustoming themselves to a position shorter than natural in spite of their slow yielding to the inadequate extending and counter extending forces,—in addition, also to other still more important facts which, as we shall prove hereafter, oblige us eventually to employ much more force in the reduction,—this method of treatment leaves the parts for a long time in the precise condition most favourable to the production of spasms. What would be said to the surgeon who would venture to recommend three days, or one or two weeks, as a proper time for continuing gradual extension or counter-extension in a case of luxation? But the advocates of such periods in the treatment of fractures contend that the application of so great a force as is necessary to produce a rapid reduction of a shortened limb, is productive of spasm from its very violence. We know not what conception these gentlemen form of the requisite force, but one thing is clear:—In dislocations generally, the force required to reduce the limb, and keep it in a state of reduction, is far greater than that which can be necessarily or legitimately employed in fractures of the neighboring bones; yet we have never seen it produce spasm. The latter force, under proper management, is always trivial in amount, except in the first adjustment of fractures with interlocking spicula, and much of the evil occasionally resulting from the apparatus employed in effecting it is chargeable, not to the force exerted, but to certain errors in manipulation, which will be discussed in this and future notes.

The first, and most important measure, then, for the relief or prevention of muscular spasm in fractures is undoubtedly the entire coaptation of the fragments as soon as possible, and proper support of the limb in the extended position, except in certain peculiar fractures, which will be hereafter mentioned. But it is also highly important to avoid undue pressure upon injured, or even uninjured muscles; for positive irritation of the latter will often produce nervous reactions provoking the spasmodic action of the former. Hence, the dressings should be as light and simple as possible, and the forces employed in retention as gentle as may be.

It is unfortunate that the terms "extending and counter-extending bands" should ever have been adopted in treating of fractures, however appropriate in speaking of luxations. "Words are things," and these words are the origin of the vast complication of screws, wheels and axles, springs, &c. &c., which disgraces this part of the materia-chirurgica. Extension should never be made except by the hands of the surgeon, and the bands should be employed simply for retention. It is certainly not surprising that spasms frequently occur under the action of instruments of torture of which the force cannot be estimated with any accuracy, and which act with a seeming surety altogether fictitious.

With regard to the time required gradually to bring even a recent fractured thigh, accompanied with decided contraction of the muscles and shortening of the limb, to its full length by very moderate force, four hours would be generally found amply sufficient, if the surgeon could give undivided attention to the case. But, as other engagements necessarily interfere with the very frequent exercise of manual extension, twenty-four hours, and three or four operations will be found an ample allowance for the least tractable cases, unless the accident has been neglected or maltreated for more than a week. There are some who contend against this position on the plea that, in fractures attended with severe laceration of the muscles, the remaining attachments may give way, and the vitality of the member be destroyed under the attempt at what they are pleased to call "such sudden extension." This idea is but another proof of the false impression with regard to the force necessary in reducing fractures accompanied with shortening of a member. If the accident alluded to could occur from the exercise of any *legitimate* degree of extension, the case is one in which there can be no longer any question of the propriety of amputation, and both the slow and the rapid adjustment of the fragments are alike impracticable. Indeed, it not unfrequently happens that no extension whatever is necessary in a very recent fracture of the thigh: we have nothing to do but to place the limb in a retentive apparatus provided with inextensible bands sufficiently strong effectually to resist the tonic contractility of the muscles; and the limb, already of the full length, in consequence of the feebleness resulting from the injury, will remain so to the end of the treatment. Of this, however, we shall speak again in a future number.

There exists, then, no reasonable objection to the early removal of the rough and sharp ends of the fragments from the false position in which they prove sources of serious irritation, or to the early elongation of the muscles when contracted, so as to remove the two most fertile sources of spasm. But there are many men of plainly practical minds who cannot readily adopt any principle, however obvious, unless it be submitted to the unnecessary test of experience, even after it has been fully demonstrated; for the satisfaction of such minds it may be well to state, that during some years of observation on very numerous cases of fractures of the femur, we have constantly observed that the number of cases of troublesome spasms was nearly in reverse proportion to the rapidity of perfect reduction; those surgeons whose efforts at extension are most protracted, meeting with a very disproportionate number of such accidents.

But the perfect coaptation of the fragments does not, in every instance, secure the patient against spasms. In some rare cases, the previous injury to the soft parts induces such contractions even after the perfect adjustment of the limb. The internal exhibition of a strong opiate, when not contra-indicated by accidental complications, the topical use of warm opiate fomentations—the watery solution being the best—and enveloping the limb in hot towels or warm bran, has very readily removed the difficulty in every such instance which we have observed.

